# Stress and Alcohol: Epidemiologic Evidence

**DOI:** 10.35946/arcr.v34.4.03

**Published:** 2012

**Authors:** KM. Keyes, ML. Hatzenbuehler, Bridget F. Grant, Deborah S. Hasin

**Affiliations:** **K.M. Keyes, Ph.D.,***is an assistant professor of epidemiology in the Department of Epidemiology, Mailman School of Public Health, Columbia University, and a data analyst at the New York State Psychiatric Institute, both in New York, New York.*; **M.L. Hatzenbuehler, Ph.D.,***is a Robert Wood Johnson Health and Society Scholar at the Center for the Study of Social Inequalities in Health, Mailman School of Public Health, Columbia University, New York, New York.*; **Bridget F. Grant, Ph.D., Ph.D.,***is chief of the Laboratory of Epidemiology and Biometry, Division of Intramural Clinical and Biological Research, National Institute on Alcohol Abuse and Alcoholism, Bethesda, Maryland.*; **Deborah S. Hasin, Ph.D.,***is a professor of clinical epidemiology in the Department of Psychiatry, College of Physicians and Surgeons, Columbia University; and a research scientist at the New York State Psychiatric Institute; both in New York, New York.*

**Keywords:** Alcohol use and abuse, alcohol use disorders, stress, stress as a cause of alcohol and other drug use, risk factors, psychological stress, stress response, coping, stressors, general life stress, catastrophe, child abuse, minority group, epidemiological indicators

## Abstract

Exposure to stress often is psychologically distressing. The impact of stress on alcohol use and the risk of alcohol use disorders (AUDs) depends on the type, timing during the life course, duration, and severity of the stress experienced. Four important categories of stressors that can influence alcohol consumption are general life stress, catastrophic/fateful stress, childhood maltreatment, and minority stress. General life stressors, including divorce and job loss, increase the risk for AUDs. Exposure to terrorism or other disasters causes population-level increases in overall alcohol consumption but little increase in the incidence of AUDs. However, individuals with a history of AUDs are more likely to drink to cope with the traumatic event. Early onset of drinking in adolescence, as well as adult AUDs, are more common among people who experience childhood maltreatment. Finally, both perceptions and objective indicators of discrimination are associated with alcohol use and AUDs among racial/ethnic and sexual minorities. These observations demonstrate that exposure to stress in many forms is related to subsequent alcohol consumption and AUDs. However, many areas of this research remain to be studied, including greater attention to the role of various stressors in the course of AUDs and potential risk moderators when individuals are exposed to stressors.

Exposure to varying forms of stress is an integral life experience that can provoke a variety of reactions. In research on alcohol, drug, and psychiatric disorders, the term “stress” often is understood to indicate any experience denoting adversity (Dohrenwend 2000). Stress exposures consist of external stimuli that are threatening or harmful; elicit fear, anxiety, anger, excitement, and/or sadness; and are negative in impact and outcome (Sinha 2001, 2008). Mild to moderate levels of stress can present challenges that are within a person’s capability to overcome, producing a sense of mastery and accomplishment that eventually result in a positive outcome. However, adverse experiences that exceed the coping abilities of the individual increase the risk for psychopathology (Lazarus 1999; Levine 2005; McEwen 2007; Selye 1976; Sinha 2008).

Just as people vary in their capabilities, stress exposures can be viewed as varying across several dimensions (see figure 1). One dimension is severity, which can range from mild (e.g., the daily hassles of family and job among healthy individuals whose basic needs are met) to severe (e.g., extreme adversity that threatens the life, physical integrity, health and home of oneself and one’s loved ones). Other dimensions, not necessarily orthogonal to each other, include whether the stressor occurred during childhood or maturity, the degree to which the stressor is acute or chronic and expected or unexpected, whether the threat is emotional or physical, and the difficulty of discerning whether the stressor was the cause or consequence of the health outcome under consideration.

This article presents evidence for the effect of four categories of stressors, including general life stress, catastrophic/fateful stress, childhood maltreatment, and minority stress, each of which encompasses a range of specific kinds of stressors (see figure 2). Each category of stressors is evaluated according to the dimensions shown in figure 1, and the extant epidemiologic evidence for the effect of each on both alcohol use and alcohol use disorders (AUDs) is reviewed.

## General Life Stressors and AUDs—Evidence From National Surveys

National surveys often include some measure of general life stress that may range from common experiences, such as moving or changing jobs, to uncommon experiences, such as severe threats to personal integrity and arrest. The severity of the events often is variable; for example, a divorce that may be stressful for some individuals can be a relief for others, and the death of a relative may refer to a parent or spouse or to a distant relative with little connection to the respondent’s day-to-day life. Nevertheless, the overall number of these experiences is related to alcohol outcomes (see table 1). In the 2001–2002 National Epidemiologic Survey on Alcohol and Related Conditions, respondents reported on 12 general life stressors, ranging from items such as changing jobs or moving, to trouble with a boss or coworker, trouble with a neighbor, and a family member in poor health, to being the victim of a crime, being unemployed or fired from a job, and divorce or breakup of a steady relationship. The data show that the number of past-year stressors experienced was related to any current drinking, current binge drinking (i.e., consuming five or more drinks for men or four or more drinks for women at least once in the past year), and current AUDs. Among men, the relationship with each alcohol outcome steadily increased from 0 to approximately 6 stressors, after which the relationship tapered off and tended to decrease at 10 or more stressors. Among women, the relationship with each outcome generally was more linear, with increases in prevalence at each increase in past-year stressors (see table 1).

Various studies in smaller adult community samples also have found that the number of general life stressors is associated with alcohol consumption and problem alcohol use (which may not necessarily meet the criteria of an AUD) (Cole et al. 1990; King et al. 2003). However, one population-based longitudinal study of older adults (mean age 61 years) did not demonstrate long-term effects (i.e., at 1 year or more after the event) of acute stressful life events on patterns of alcohol consumption (Skaff et al. 1999). A national prospective study of 3,006 women found an increased risk of alcohol abuse after being an assault victim, with no evidence of reverse causation (i.e., that alcohol consumption alone contributed to the risk for assault) (Kilpatrick et al. 1997).

However, other studies have indicated that excessive alcohol use also increases the risk for sexual assault (Abbey et al. 1994; Corbin et al. 2001); therefore, the relationship between assault and alcohol use likely is bidirectional. Finally, several general population studies have found an increase in the incidence of AUDs following job loss, particularly among men (Catalano et al. 1993; Crawford et al. 1987). It is noteworthy, however, that the context of a job loss likely is important for its impact on the risk of AUDs. For example, the meaning of the lost job may be different for a worker whose plant is shut down after he or she has worked for 30 years in the same position compared with an artist or a musician accustomed to temporary work. Nevertheless, these studies indicate that any type of job loss is associated with increased risk of AUDs.

Genetic factors may influence the relationship between exposure to general stressors and alcohol and other drug use. In a longitudinal study of 295 college students who for 2 years provided daily reports of stressful events as well as alcohol and drug use via the internet, those who carried two copies of a specific variant in regulatory region of the gene encoding a protein involved in the actions of the brain signaling molecule serotonin (i.e., who were homozygous for the *s* allele of 5-HTTLPR serotonin transporter promoter) were at substantially increased risk for heavy drinking and drug use if they experienced a high level of stressful life events compared with students carrying only one or no copy of this allele (Covault et al. 2007).

It also is important to note that daily exposure to interpersonal stress, such as problems at work, trouble with the police, or breakup of romantic relationships also may be influenced by having an AUD. Although these exposures likely are stressful for anyone experiencing them, they can be as much a consequence as a cause of an AUD. Therefore, teasing apart the temporal and causal directions of relationships between these adult stressors and alcohol use is a difficult task in general-population epidemiologic samples.

## Fateful/Catastrophic Events and AUDs

With respect to the various correlated dimensions of stress in human populations described earlier, fateful/catastrophic events, such as direct exposure to a disaster or terrorism attack, typically lie on the more extreme end of the severity continuum. These stressors usually are acute and unexpected, and exposure is very unlikely to result from an individual’s alcohol consumption. However, the “fatefulness” of the event may depend on the specific circumstances of the event. For example, studies of people exposed to nightclub disasters (e.g., from fires and terrorist attacks) (Kennedy et al. 2005; Mahoney et al. 2005) involve individuals who are younger and more likely to consume alcohol than the general population. The study of such events still may provide important information, but the type of individuals involved and the appropriate control group must be considered carefully. Fateful/catastrophic events can involve both physical threat to one’s life and emotional threat (e.g., knowing someone lost or killed in the fateful/catastrophic incident, fear of additional exposures) and generally can occur at any point in the life course.

Both in the United States and internationally, many studies have addressed the relationship between different types of natural and man-made disasters and alcohol consumption, including studies of exposure to natural disasters, such as flooding (North et al. 2004), volcano eruptions (Adams and Adams 1984), earthquakes (Shimizu et al. 2000), and hurricanes (Cerda et al. 2011; Kohn et al. 2005). Studies also have investigated the consequences of exposure to man-made disasters, such as mass shootings (North et al. 1994; Smith et al. 1999), fire or grotesque death (Green et al. 1985; Reijneveld et al. 2003; Sims and Sims 1998), ferry disasters (Joseph et al. 1993), and nuclear accidents (Kasl et al. 1981). Studies covering a time-frame of a year or less after the disaster consistently have indicated postdisaster increases in alcohol consumption (Joseph et al. 1993; Kasl et al. 1981; Kohn et al. 2005; Reijneveld et al. 2003; Sims and Sims 1998; Smith et al. 1999). Studies with multiple and/or longer followups generally have found attenuation of this relationship over time (Joseph et al. 1993).

Several studies also have addressed alcohol consumption in response to exposure to terrorism. Substantial research on mental health in general and alcohol consumption specifically has been conducted after the terrorist attacks on the World Trade Center in New York City and the Pentagon in Washington, DC, on September 11, 2001 (9/11). These studies have indicated that alcohol consumption generally increased in both New York City and elsewhere in the short term following the attacks. Thus, increased alcohol use was found among the following groups:

- Survivors of the attack on the Pentagon (Grieger et al. 2003);
- Residents of Manhattan in the one month and/or six months following the attack (Ho et al. 2002; Vlahov et al. 2002, 2004);
- Residents in the tri-State area of Connecticut, New York, and New Jersey (Melnik et al. 2002); and
- Adults from a nationally representative sample (Stein et al. 2004).

Longer-term studies showed increased alcohol consumption 1 and 2 years later among New Yorkers at greater exposure levels to the attack (Boscarino et al. 2006).

Few studies have examined alcohol use and terrorism exposure outside the United States, but two studies of adolescents in different cities in Israel found that geographic proximity to terrorist attacks was associated with greater quantity and frequency of drinking as well as with binge drinking (Schiff et al. 2006, 2007).

Several studies have been able to control for predisaster drinking levels, the lack of which had been a limitation of most of the aforementioned epidemiologic research. These studies have documented an increase in alcohol consumption following exposure to disaster independent of the consumption levels measured prior to the exposure (Cerda et al. 2011; Hasin et al. 2007*a*; Richman et al. 2004). A recent meta-analysis of 27 studies assessing substance use in response to terrorism that included studies with follow-up times ranging from 1 week to more than 2 years found a pooled effect indicating that the population level of alcohol consumption is increased following a terrorist attack (DiMaggio et al. 2009).

The research described above focuses on any alcohol consumption after disaster. Studies of AUDs and problem drinking following major disasters have been less consistent. Following the Oklahoma City bombings in 1995, North and colleagues reported no increase in incident AUDs, either in survivors of the attack (North et al. 1999) or in rescue workers (North et al. 2002). Survivors of other disasters, such as Hurricane Andrew (David et al. 1996), flooding (Green et al. 1992; North et al. 2004), and jet crashes (Smith et al. 1990), as well as a combined sample of survivors from the Oklahoma City terrorist bombing and the bombing of the U.S. embassy in Nairobi, Kenya (North et al. 2005) also showed no evidence of increases in incident AUDs. Studies assessing the impact of 9/11 found that neither living near the attack site nor knowing someone lost or killed was associated with incident alcohol problems 6 months following the attack (Vlahov et al. 2006); moreover, exposure to 9/11 was not associated with the trajectory of alcohol use and binge drinking in the 3 years following the attack (Cerda et al. 2008). In a recent pooled analysis of data from 10 different disasters, including exposure to flooding, shootings, and plane crashes, North and colleagues (2010) again reported no evidence of increased risk for incident AUDs after these events, although people with pre-existing AUDs were more likely to report increased drinking after these events.

Several studies contradict the above evidence, however, as follows:

- Evidence from survivors of Hurricane Katrina indicates elevated rates of alcohol problems compared with national and local predisaster averages (Flory et al. 2009). Furthermore, increases in binge drinking were found among those most exposed to the hurricane, controlling for prehurricane alcohol use (Cerda et al. 2011).
- Among New Yorkers interviewed at 1 and 2 years after 9/11, greater exposure levels predicted binge drinking at 1 year but not 2 years and an increase in alcohol dependence at both time points (Boscarino et al. 2006).
- Seven months after the Mount St. Helens volcano eruption, alcohol-center referrals and liquor-law violations had increased compared with the pre-eruption period (Adams and Adams 1984).
- Survivors of the Beverly Hills Supper Club fire seemed to have an increase in alcohol abuse more than 2 years after the fire (Green et al. 1985).

Thus, the literature is inconsistent on the role of fateful traumatic events in the development of AUDs. It is noteworthy, however, that studies of incident AUDs after major disasters were conducted in adult populations in which the incidence of such disorders generally is low (Hasin et al. 2007*b*). Studies of incident AUD risk following exposure to disaster in adolescent and young adult populations are necessary to comprehensively understand the relation between disaster and incident AUDs.

A substantial literature also has documented increased alcohol consumption and risk for AUDs among war veterans, especially those exposed to active combat (Hoge et al. 2006; Jacobson et al. 2008; Milliken et al. 2007; Shipherd et al. 2005). Causal inference from this literature is complicated, however, because people who perform military duty most often are young men at high baseline risk for AUDs. In addition, exposure to combat is not randomly assigned, and people who have sensation-seeking personality characteristics are more likely to both be assigned to combat and, independently, develop AUDs.

## Child Maltreatment and AUDs

Childhood maltreatment includes many adverse exposures (e.g., sexual, emotional, and/or physical abuse and emotional and/or physical neglect) during the first 18 years of life. With respect to the various correlated dimensions of stress in human populations described earlier, childhood maltreatment experiences range from mild (e.g., occasionally saying hurtful things) to severe (e.g., chronic physical and/or sexual abuse). Although these stressors can be acute, they often are chronic throughout childhood; furthermore, they are very unlikely to be a consequence of alcohol consumption as they typically occur before drinking initiation. Childhood maltreatment can involve both physical threat (e.g., physical and sexual abuse or physical neglect of needs) and emotional threat (e.g., emotional abuse and neglect). These experiences are common and may account for a significant proportion of all adult psychopathology (Afifi et al. 2008; Green et al. 2010). Further, events frequently co-occur (Dong et al. 2004; Dube et al. 2002; Edwards et al. 2003; Finkelhor et al. 2007)—in other words, exposure to one type of childhood maltreatment increases the risk of exposure to others.

Epidemiologic studies addressing the impact of adverse childhood events on alcohol consumption and AUDs have employed several types of designs, including cross-sectional studies of adults with retrospective assessment of adverse childhood events, prospective cohort studies, and studies of twin and other genetically informative samples. Studies generally have shown that most forms of child maltreatment are related to higher risk of adolescent alcohol consumption (Bensley et al. 1999; Hussey et al. 2006; Sartor et al. 2007; Thornberry et al. 2001) and adult alcohol consumption and AUDs (Anda et al. 2002; MacMillan et al. 2001; Molnar et al. 2001; Nelson et al. 2006). One review documented that childhood maltreatment and other childhood stressors were associated with earlier onset of adolescent alcohol consumption and with AUDs in adulthood (Enoch 2010).

Childhood maltreatment is more likely to occur among children of alcoholics (Gilbert et al. 2009); in these cases, the parents may not only engage in harmful parenting practices (Kettinger et al. 2000; Stanger et al. 2004; Suchman et al. 2007, 2008) but also may pass along genes increasing the risk of AUDs to their offspring. Thus, the specificity of the relationship between maltreatment and alcohol use in the context of these other risk factors remains an open debate. Furthermore, psychiatric comorbidity also may confound the relationship between early maltreatment and AUDs because maltreatment affects the risk for multiple psychiatric disorders (Green et al. 2010; Kendler et al. 2000; Kessler et al. 1997; Widom et al. 2007*a*), and AUDs are highly comorbid with other forms of psychopathology (Hasin et al. 2007*b*). Studies using animal models, which can control for environmental factors and comorbidity, have suggested that extended stress in early life leads to later self-administration of alcohol (Cruz et al. 2008; Miczek et al. 2008). However, some epidemiologic studies suggest that the relationship between maltreatment and AUDs may be at least partially confounded by family history of alcohol problems. For example, a prospective cohort study that compared court-recorded cases of abuse and neglect with matched community controls in the Midwest found no remaining association between early abuse and adult AUDs^1^ after controlling for family history of alcohol problems among men (Widom et al. 1995, 2007*b*); only among women physical neglect remained associated with AUDs.

However, several studies that controlled for family history of alcoholism have indicated a persistent relationship between childhood adverse events, including parental divorce (Pilowsky et al. 2009; Thompson et al. 2008) and death of a parent or foster home placement (Kendler et al. 1996; Pilowsky et al. 2009), and adult risk for AUDs. Another study documented strong and significantly increased odds of AUDs based on retrospective assessment of childhood sexual abuse among same-sex twins in Australia (Nelson et al. 2002), even after controlling for family background variables such as parental alcohol problems. Finally, recent data from a population-based study of twins in Virginia reported that participants who reported any maltreatment were 1.74 times as likely to experience an AUD in adulthood as were people who did not report maltreatment, and although controlling for family-level risk factors substantially attenuated the observed association, a direct effect remained after control (Young-Wolff et al. 2011).

Research now is examining specific genetic variations (i.e., polymorphisms) as moderators of the relationship between child maltreatment and AUDs. The finding that functional polymorphisms in the gene encoding the monoamine oxidase A enzyme (MAOA) (Caspi et al. 2002) interact with childhood maltreatment to predict antisocial behavior in adulthood stimulated research on whether this effect generalizes to substance use disorders; however, thus far, the findings could not be replicated (Young et al. 2006). Other studies have focused on the previously mentioned serotonin transporter promoter variant, 5-HTTLPR, and its interaction with stressful experiences in a wide variety of psychiatric outcomes after researchers detected such an interaction for major depression (Caspi et al. 2003). This DNA sequences exists in two alleles, *l* and *s* alleles; thus, a person can carry either two *l* or two *s* alleles (i.e., be homozygous for *l* or *s*) or one *l* and one *s* allele (i.e., be heterozygous). One study found that youth with court-documented maltreatment were at higher risk for early-onset alcohol use if they had the heterozygous (*s/l*) genotype compared with the *l/l* genotype (Kaufman et al. 2007). In another youth study, the effect of the same heterozygous genotype on increased risk for substance use was attenuated in families providing involved-supportive parenting (Brody et al. 2009*a*). In an innovative approach involving random assignment of the environment, the investigators then randomized at-risk families to an intervention designed to increase involved-supportive parenting or a control condition (Brody et al. 2009*b*). Among those with the heterozygous 5-HTTLPR genotype, children in treated families had less substance use at followup compared with children of the control families (Brody et al. 2009*b*). Taken together, these studies suggest that the risk for later alcohol outcomes is affected by an interaction of stressful early home environments and genetic vulnerability.

## Minority Stress and AUDs

Minority stress is defined as exposure to specific stressors that result from a person’s minority status, especially prejudice and discrimination events (Meyer 2003*b*; Williams et al. 2003). These events range from mild (e.g., daily hassles, such as being followed in a store) to more severe (e.g., being a victim of a violent crime) and include both emotional (e.g., workplace harassment [Waldo 1999]) and physical (e.g., hate crimes [Herek 2009]) threats to self. Minority status cannot be attributed to having an AUD, making one aspect of interpretation straightforward in studies in this area. Although minority stress can involve acute events, it most frequently is viewed as a chronic exposure that occurs across the entire life course (Williams et al. 2003). Finally, minority stressors vary with respect to whether they are expected. Research has indicated that although many stressors that members of minority groups confront are unanticipated, one consequence of repeated exposure to discrimination is that people begin to expect rejection based on their stigmatized identity (Mendoza-Denton et al. 2002).

### Racial/Ethnic Minorities

According to minority stress models, the stress resulting from prejudice and discrimination should lead to elevations in alcohol use among minority group members. Patterns of alcohol use among racial/ethnic minorities, however, fail to correspond to these predictions. Although Native Americans have higher rates of alcohol consumption and AUDs compared with non-Hispanic Whites (Hasin et al. 2007*b*), several large surveys have indicated lower rates of alcohol consumption and AUDs among non-Hispanic Blacks, Asians, and Hispanics compared with Whites (Breslau et al. 2006; Hasin et al. 2007*b*; Kessler et al. 1994). These minority groups also have lower rates of other psychiatric disorders (e.g., major depression), leading to what has been called the “minority paradox” (Williams 2001) in mental health research—that is, minority groups such as Blacks and Hispanics have lower rates of psychiatric and substance disorders despite greater exposure to institutional and interpersonal discrimination that has been shown to engender substantial stress via biological (Lewis et al. 2006) and psychological (Hatzenbuehler 2009) mechanisms. In contrast to these findings from between-group studies, within-group studies consistently show that perceived discrimination is associated with alcohol outcomes. This association has been found in Blacks (McLaughlin et al. 2010*b*; Taylor and Jackson 1990; Yen et al. 1999), Filipino Americans (Gee et al. 2007) and Asian-American adolescents (Yoo et al. 2010).

### Sexual Minorities

In contrast to racial/ethnic minorities, lesbian, gay, and bisexual (LGB) individuals have higher rates of substance use and substance use disorders than their heterosexual peers (Garofalo et al. 1998; Russell et al. 2002; Ziyadeh et al. 2007); this difference applies to both adolescents (Eisenberg and Wechsler 2003; Hatzenbuehler et al. 2008) and adults (Burgard et al. 2005; Cochran et al. 2000; Drabble et al. 2005). Although research has tended to primarily examine perceived discrimination as a risk factor for internalizing psychopathology, such as depression and anxiety, recent studies also have shown higher levels of alcohol use (Hatzenbuehler et al. 2011) and AUDs (McCabe et al. 2010) among LGBs who perceive that they have experienced higher levels of discrimination.

Because of their design, these studies cannot rule out reverse causality—that is, that individuals with alcohol problems may perceive and report greater discrimination. In order to address some of these methodological limitations of subjective measures of discrimination, recent studies have developed novel measures for operationalizing objective stressors that LGB individuals confront, including institutional forms of discrimination (e.g., anti-marriage laws or employment discrimination policies). Because these institutional stressors occur outside the control of LGB individuals, they are not confounded with mental health status and therefore provide a stronger test of the effect of discrimination on mental health than measures of subjective stress. Studies are beginning to document the relationship between these objective stressors and LGB health, including alcohol use. For example, a recent study examined the impact of State-level ballot initiatives banning gay marriage on the prevalence of psychiatric and substance use disorders in LGB populations (Hatzenbuehler et al. 2010). The results indicated that LGB respondents living in States that passed such bans in 2004 had significantly greater increases in psychiatric disorders and AUDs than did LGB respondents in States that did not pass such bans (Hatzenbuehler et al. 2010). This research demonstrates the potential importance of incorporating more objectively-defined indices of social stress into research on alcohol use among minority populations. Indeed, an examination of how and why such social stressors contribute to the development and maintenance of AUDs within LGB populations represents a crucial avenue for future inquiry.

## Conclusion

The psychological and psychiatric effects of stress remain an important mechanism for individual differences in all areas of mental health. Substantial evidence exists that fateful/catastrophic events, such as exposure to disaster and terrorism; childhood adversities, such as maltreatment; interpersonal stressors, such as divorce and job loss; and chronic minority stress affect alcohol consumption and AUDs. Although these data demonstrate the importance of stress in the development of alcohol problems in human populations, substantial work remains to be done in these areas. Refined measures of stress exposures; careful assessment of confounding and reverse causation; an examination of AUD course, including relapse; and the potentiating of stress effects by genetic vulnerability, personality factors, macro-social factors, and other important biological and social domains remain important topic areas in need of more epidemiologic study. Exploring the epidemiology of stress in human populations can help integrate and translate work in experimental human and animal models in order to demonstrate the real-world effects of these common yet often devastating exposures on alcohol use and misuse.

## Figures and Tables

**Figure 1:**
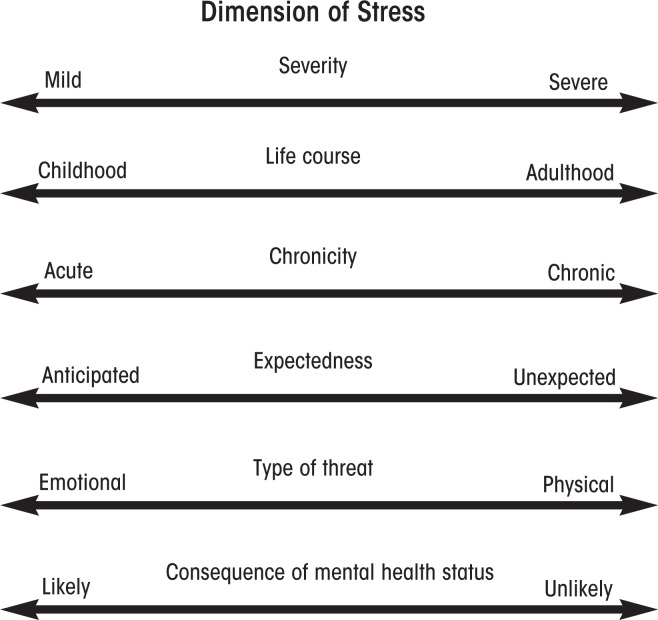
Dimensions of stressful experiences.

**Figure 2:**
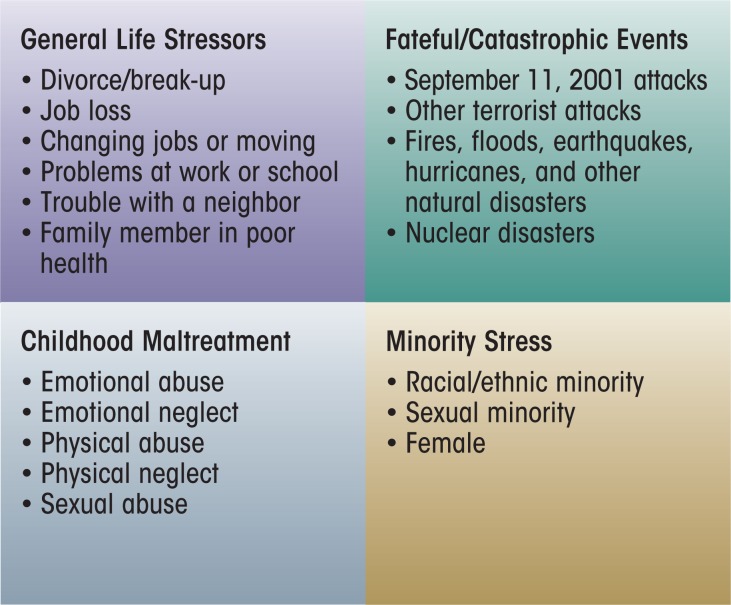
Four categories of stressors and examples of exposures within each stress category.

**Table 1:** Relationship Between Number of Past-Year Stressors and Prevalence of Current Drinking, Current Binge Drinking, and Current Alcohol Use Disorders Among Men and Women in the 2001–2002 National Epidemiologic Survey on Alcohol and Related Conditions (*N* = 43,093).

	Men			Women		
	**Current Drinking (% respondents)**	**Current Binge Drinking (% respondents)**	**Current Alcohol Use Disorders (% respondents)**	**Current Drinking (% respondents)**	**Current Binge Drinking (% respondents)**	**Current Alcohol Use Disorders (% respondents)**
Number of past-year stressors						
0	65.9	32.0	6.1	49.0	11.9	1.8
1	70.7	41.2	9.8	58.5	13.8	3.3
2	72.8	42.7	12.0	61.6	17.7	4.7
3	77.8	52.3	18.3	68.7	24.5	7.0
4	79.0	60.8	24.6	73.8	28.8	11.5
5	84.1	61.5	30.3	74.6	33.5	11.9
6	87.7	66.1	35.0	77.6	39.2	13.7
7	87.3	69.5	35.8	76.9	36.5	21.2
8	85.6	70.7	35.1	84.0	47.7	23.9
9	96.8	66.9	56.3	86.9	46.1	33.2
10+	66.0	65.2	36.4	89.2	50.9	40.8

SOURCE: National Epidemiologic Survey on Alcohol and Related Conditions

## Footnotes

1 AUDs in this study were defined according to the American Psychiatric Association’s *Diagnostic and Statistical Manual of Mental Disorders, Third Edition, Revised* (DSM–III–R).
